# Association of a Body Mass Index Genetic Risk Score with Growth throughout Childhood and Adolescence

**DOI:** 10.1371/journal.pone.0079547

**Published:** 2013-11-11

**Authors:** Nicole M. Warrington, Laura D. Howe, Yan Yan Wu, Nicholas J. Timpson, Kate Tilling, Craig E. Pennell, John Newnham, George Davey-Smith, Lyle J. Palmer, Lawrence J. Beilin, Stephen J. Lye, Debbie A. Lawlor, Laurent Briollais

**Affiliations:** 1 School of Women’s and Infants’ Health, The University of Western Australia, Perth, Western Australia, Australia; 2 Samuel Lunenfeld Research Institute, University of Toronto, Toronto, Ontario, Canada; 3 MRC Centre for Causal Analyses in Translational Epidemiology, School of Social and Community Medicine, University of Bristol, Bristol, United Kingdom; 4 School of Social and Community Medicine, University of Bristol, Bristol, United Kingdom; 5 Ontario Institute for Cancer Research, University of Toronto, Toronto, Ontario, Canada; 6 School of Medicine and Pharmacology, The University of Western Australia, Perth, Western Australia, Australia; University of Antwerp, Belgium

## Abstract

**Background:**

While the number of established genetic variants associated with adult body mass index (BMI) is growing, the relationships between these variants and growth during childhood are yet to be fully characterised. We examined the association between validated adult BMI associated single nucleotide polymorphisms (SNPs) and growth trajectories across childhood. We investigated the timing of onset of the genetic effect and whether it was sex specific.

**Methods:**

Children from the ALSPAC and Raine birth cohorts were used for analysis (n = 9,328). Genotype data from 32 adult BMI associated SNPs were investigated individually and as an allelic score. Linear mixed effects models with smoothing splines were used for longitudinal modelling of the growth parameters and measures of adiposity peak and rebound were derived.

**Results:**

The allelic score was associated with BMI growth throughout childhood, explaining 0.58% of the total variance in BMI in females and 0.44% in males. The allelic score was associated with higher BMI at the adiposity peak (females  =  0.0163 kg/m^2^ per allele, males  =  0.0123 kg/m^2^ per allele) and earlier age (-0.0362 years per allele in males and females) and higher BMI (0.0332 kg/m^2^ per allele in females and 0.0364 kg/m^2^ per allele in males) at the adiposity rebound. No gene:sex interactions were detected for BMI growth.

**Conclusions:**

This study suggests that known adult genetic determinants of BMI have observable effects on growth from early childhood, and is consistent with the hypothesis that genetic determinants of adult susceptibility to obesity act from early childhood and develop over the life course.

## Introduction

Twin and family studies have provided evidence that body mass index (BMI) is strongly heritable [1], [2], [3], [4]. Recent genome-wide association studies (GWAS) have begun to uncover genetic loci contributing to increases in BMI in adulthood [5], [6], [7], [8], [9], [10], [11]. The largest genome-wide meta-analysis of BMI published to-date included 249,796 individuals from the Genetic Investigation of Anthropometric Traits (GIANT) Consortium; which confirmed 14 previously-reported loci and identified 18 novel loci for BMI [5]. There has been one GWAS to date that has focused on a dichotomous indicator of childhood obesity [12], but none looking at BMI on a continuous scale in childhood.

Once adult height is attained, changes in BMI are largely driven by changes in weight. In contrast, during childhood and adolescence, changes in BMI are influenced by both changes in height and weight. Therefore, genetic variants that affect adult BMI may influence change in weight, height or both during childhood. Previous studies of adult BMI single nucleotide polymorphisms (SNPs) in relation to infant and child change in growth have shown little evidence of an association with birth weight [13], [14], [15], but have shown evidence that these loci are associated with more rapid height and weight gain in infancy [13], [15], and higher BMI and odds of obesity at multiple ages across the life course [13], [14], [15], [16], [17].

BMI growth over childhood and adolescence is complex; children tend to have rapidly increasing BMI from birth to approximately 9 months of age where they reach their adiposity peak, BMI then decreases until about the age of 5-6 years at adiposity rebound and then steadily increases again until just after puberty where it tends to plateau through adulthood. The BMI and timing at the adiposity peak [18] and adiposity rebound [19], [20], [21], [22], [23], [24] have been shown to be associated with later BMI. Genetic variants could also affect features of the growth trajectory and shape key developmental milestones, including the adiposity peak [25], adiposity rebound, and onset of puberty between 10 and 13 years [17], [26], [27], [28]. Sovio et al [17] and Belsky et al [15] have recently shown that SNPs associated with adult BMI are also associated with earlier age and higher BMI at adiposity rebound. Genetic influences on the adiposity peak remain poorly understood. Understanding whether and how genetic loci are associated with BMI and other anthropometric measures differentially across the life course may shed light on the biological pathways involved, as well as insights into the development of obesity to inform the design of interventions.

To date, there has been no comprehensive study of how all known genetic variants of adult BMI influence growth over childhood and adolescence (BMI, height and weight) and related growth parameters (age and BMI at the adiposity peak and rebound). One of the limitations of previous studies is they have not stratified by sex, despite some evidence that sex-specific differences in body composition may be partly due to genetics [29], [30]. Therefore, in the current study we:

1. Examine the association between an allelic score of 32 adult BMI associated alleles and BMI, weight and height growth trajectories from birth to age 17 in two birth cohorts.
2. Assess whether the association between BMI trajectories and the 32 individual genetic loci are sex specific.

## Materials and Methods

### Study Populations

**ALSPAC.** The Avon Longitudinal Study of Parents and Children (ALSPAC) is a prospective cohort study. The full study methodology is published elsewhere [31] (www.bristol.ac.uk/alspac). Pregnant women resident in one of three Bristol-based health districts with an expected delivery date between 1 April 1991 and 31 December 1992 were invited to participate. Invitation cards indicated that study consent was ‘opt out’, i.e. women not actively declining participation would be included in future data collection follow-up. Follow-up included parent and child completed questionnaires, links to routine health care data, and clinic attendance. 7,868 individuals were included in this study based on the following criteria: at least one parent of European descent, live singleton birth, unrelated to anyone in the sample, no major congenital anomalies, genotype data, and at least one measure of BMI throughout childhood. Ethical approval for the study was obtained from the ALSPAC Law and Ethics Committee and the Local Research Ethics Committees.

**Raine.** The Western Australian Pregnancy Cohort (Raine) Study [32], [33], [34] is a prospective pregnancy cohort where 2,900 mothers were recruited prior to 18-weeks’ gestation between 1989 and 1991 (http://www.rainestudy.org.au/). 1,460 individuals were included in this study using the same criteria as in the ALSPAC cohort. The study was conducted with appropriate institutional ethics approval from the King Edward Memorial Hospital and Princess Margaret Hospital for Children ethics boards, and written informed consent was obtained from all mothers.

BMI was calculated from weight and height measurements in both cohorts. Additional information on the measurements in each cohort is provided in the supplementary material (see Methods S1). Access to data and associated protocols from the two cohorts needs to follow the cohort guidelines outlined on their respective websites.

### Genotyping and allelic score

Imputed genotypic data used in both cohorts has been previously described [35], [36] (details in supplementary material; see Methods S1). Speliotes et al [5] reported 32 variants to be associated with BMI, while Belsky et al [15] selected a tag SNP from each LD block that had previously been shown to be associated with BMI-related traits. We selected 32 SNPs that were from either of these two manuscripts; SNPs reported in these two manuscripts that were within the genes of interest were all in high LD (r^2^>0.75) so one loci was selected to be included. All SNPs imputed well (all R^2^ for imputation quality > 0.7, mean = 0.981), therefore, dosages from the imputed data were used (i.e. the estimated number of increasing BMI alleles). An ‘allelic score’ was created by summing the dosages for the BMI-increasing alleles across all 32 SNPs [37]. A sensitivity analysis was conducted whereby the alleles were weighted by the published effect size for adult BMI. The weighted score gave the same conclusions as the unweighted score; therefore only the unweighted score is presented.

### Longitudinal Modelling and derivation of growth parameters

Modelling BMI longitudinally from birth throughout childhood is complex due to the two inflection points, adiposity peak in infancy and adiposity rebound in childhood, and the increasing variance in BMI throughout childhood. For this reason the longitudinal models focused on data between 1 (when most individuals will be post adiposity peak) and 17 years of age. A semi-parametric linear mixed model, using smoothing splines to yield a smooth growth curve estimate, was fitted to the BMI, weight and height measures [38]. The basic model for the j^th^ individual and at the t^th^ time-point is as follows:
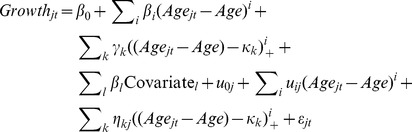



Where Growth is BMI, weight or height, Age is the mean age over the *t* time points in the sample (i.e. 8 years), κ_k_ is the *k*-th knot and (t − κ_k_)_+_  =  0 if t ≤ κ_k_ and (t − κ_k_) if t > κ_k_, which is known as the truncated power basis that ensures smooth continuity between the time windows and Covariate are the study specific (time independent) covariates. Three knot points were used, placed at two, eight and 12 years, with a cubic slope for each spline in the BMI and height models; this model provided the best fit of the data compared to other approaches [38]. The weight model had the same placement for the knots but a linear spline from 1−2 years, cubic slope for 2−8 years and 8−12 years and finally a quadratic slope for over 12 years provided a better fit to the data based having the lowest Akaike Information Criterion (AIC). All models assumed a continuous autoregressive of order 1 correlation structure.

Age and BMI at adiposity rebound were derived by setting the first derivative of the fixed and random effects from the BMI model between 2 and 8 years of age for each individual to zero (i.e. the minimum point in the curve). In addition, a second model was fit in the ALSPAC cohort only, between birth and 5 years to derive the adiposity peak; individuals with greater than 2 measures throughout this period were included [18], with 93% of included individuals having at least one measure of BMI between six and 12 months. Adiposity peak was derived by setting the first derivative of the fixed and random effects between birth and 2.5 years to zero.

### Statistical Analysis

Implausible height, weight and BMI measurements (> 4SD from the mean for sex and age specific category) were considered as outliers and were recoded to missing. Genetic differences in the trajectories were estimated by including an interaction between all components of the spline function for age and the genetic variants. The association between the allelic score and birth measures was analysed using linear regression, adjusting for gestational age at birth. Linear regression was used to investigate the associations of the allelic score with age and BMI at adiposity peak and adiposity rebound. In addition, we used the data from the final follow-up in each of the cohorts (15−17 years) to investigate, with linear regression, the association between the adiposity peak and adiposity rebound parameters with final BMI.

The growth data were collected using three measurement sources in the ALSPAC cohort; clinic visits, routine health care visits, and parental reports in questionnaires. Trajectory analyses in ALSPAC adjusted for a binary indicator of measurement source (parent reports versus clinic/health care measurements) as a fixed effect to allow for differential measurement error. To assess population stratification, principal components generated in the EIGENSTRAT software [39]. These components revealed no obvious population stratification and genome-wide analyses with other phenotypes indicate a low lambda in the ALSPAC cohort; however in the Raine cohort there was evidence of stratification so all analyses were adjusted for the first five principal components.

*FTO* is the most replicated SNP for BMI, with the largest effect size of the BMI-associated SNPs found to date, and has been shown previously to effect childhood growth [16], [17]. We therefore repeated the analysis adjusting for the *FTO* locus. All results remained unchanged indicating that the associations between growth and the allelic score were not driven exclusively by the *FTO* effect (data not shown).

We calculated the percentage of variation in BMI explained by the allelic score at each time point in the ALSPAC cohort using the residual sums of squares from the longitudinal BMI growth model [40]. We did not calculate this in the Raine cohort as the sample size was too small for accurate estimates.

Results from the two cohorts were meta-analysed. For the allelic score analyses, a fixed-effects inverse-variance weighted meta-analysis was conducted using the beta coefficients and standard errors from the two studies. No heterogeneity using Cochran’s Q was detected between the cohorts (all P>0.05). The allelic score was considered statistically associated with the growth parameter if the P-value for the meta-analysis was less than 0.05. For the analyses of the individuals SNPs with BMI, a P-Value meta-analysis was conducted on the likelihood-ratio test (LRT) P-Values from the two studies, without weighting, and a Bonferroni significance threshold of 0.0016 was used to declare a statistically significant association. All analyses were conducted in *R* version 2.12.1 [41], using the Spida library to estimate the spline functions, the rmeta library for the effect-size meta-analysis and the MADAM library for the P-Value meta-analysis.

## Results

ALSPAC children had more BMI measures throughout childhood than the Raine children with a median of 9 (interquartile range 5−12) and 6 (interquartile range 5−7) measures, respectively (Table 1). The minor allele frequency (MAF) for the 32 SNPs ranged from 0.04 to 0.49 (Table 2). The *FTO* loci had the largest effect on adult BMI, with an effect size of 0.39, while the effect size on adult BMI for the majority of the remaining loci ranged from 0.06 to 0.2. All of the following results are reported from the meta-analysis of the two cohorts, unless otherwise specified.

**Table 1:** Phenotypic characteristics of the two birth cohorts used for analysis.

	Age Stratum	ALSPAC		Raine	
	(years)	(n = 7,868)		(n = 1,460)	
Sex [% male (N)]		7,868	51.25% (4,032)	1,460	51.58% (753)
		N	Mean (SD)	N	Mean (SD)
Number of BMI measures per person		--	8.75 (4.58)	--	5.94 (1.52)
Age	1−1.49	2,832	1.18 (0.18)	1,326	1.15 (0.09)
(years)	1.5−2.49	7,113	1.76 (0.25)	387	2.14 (0.13)
	2.5−3.49	2,537	2.95 (0.28)	956	3.09 (0.09)
	3.5−4.49	6,915	3.77 (0.23)	20	3.69 (0.17)
	4.5−5.49	1,843	5.05 (0.33)	3	5.28 (0.14)
	5.5−6.49	3,848	5.90 (0.24)	1,269	5.91 (0.17)
	6.5−7.49	2,861	7.31 (0.30)	42	7.25 (0.38)
	7.5−8.49	3,975	7.74 (0.33)	1,040	8.02 (0.27)
	8.5−9.49	4,443	8.71 (0.22)	204	8.60 (0.12)
	9.5−10.49	6,777	9.94 (0.29)	303	10.44 (0.08)
	10.5−11.49	4,917	10.75 (0.23)	926	10.64 (0.15)
	11.5−12.49	5,240	11.82 (0.21)	4	11.91 (0.36)
	12.5−13.49	6,797	12.97 (0.22)	9	13.28 (0.17)
	13.5−14.49	4,690	13.89 (0.17)	1,196	14.06 (0.17)
	14.5−15.49	2,339	15.32 (0.15)	24	14.69 (0.17)
	15.5−16.49	1,645	15.72 (0.22)	2	16.16 (0.19)
	>16.5	90	16.83 (0.24)	976	17.05 (0.24)
BMI	1−1.49	2,832	17.42 (1.51)	1,326	17.11 (1.39)
(kg/m^2^)	1.5−2.49	7,113	16.82 (1.49)	387	15.97 (1.19)
	2.5−3.49	2,537	16.48 (1.40)	956	16.14 (1.23)
	3.5−4.49	6,915	16.25 (1.39)	20	15.92 (1.41)
	4.5−5.49	1,843	16.02 (1.70)	3	15.94 (1.43)
	5.5−6.49	3,848	15.71 (1.87)	1,269	15.82 (1.62)
	6.5−7.49	2,861	16.10 (1.98)	42	16.41 (2.43)
	7.5−8.49	3,975	16.31 (2.01)	1,040	16.83 (2.38)
	8.5−9.49	4,443	17.15 (2.40)	204	16.90 (2.44)
	9.5−10.49	6,777	17.67 (2.81)	303	18.91 (3.34)
	10.5−11.49	4,917	18.25 (3.10)	926	18.55 (3.16)
	11.5−12.49	5,240	19.04 (3.35)	4	16.78 (2.64)
	12.5−13.49	6,797	19.64 (3.35)	9	21.11 (3.75)
	13.5−14.49	4,690	20.31 (3.45)	1,196	21.39 (4.02)
	14.5−15.49	2,339	21.28 (3.48)	24	21.66 (4.23)
	15.5−16.49	1,645	21.41 (3.51)	2	20.14 (3.26)
	>16.5	90	22.47 (3.40)	976	23.01 (4.28)
Birth Weight (kg)	Males	3,001	3.52 (0.53)	752	3.42 (0.57)
	Females	2,855	3.40 (0.47)	707	3.31 (0.55)
Birth Length (cm)	Males	3,001	51.13 (2.40)	675	50.12 (2.34)
	Females	2,855	50.41 (2.28)	616	49.31 (2.28)
Gestational Age (wks)	Males	3,001	39.52 (1.64)	753	39.42 (1.99)
	Females	2,855	39.65 (1.58)	707	39.42 (2.06)
BMI at Adiposity	Males	4,030	18.03 (0.76)	--	--
Peak (kg/m^2^)	Females	3,792	17.45 (0.69)	--	--
Age at Adiposity Peak	Males	4,030	8.90 (0.33)	--	--
(months)	Females	3,792	9.36 (0.49)	--	--
BMI at Adiposity	Males	3,642	15.62 (1.04)	697	15.53 (0.93)
Rebound (kg/m^2^)	Females	3,225	15.53 (1.06)	647	15.42 (0.95)
Age at Adiposity	Males	3,642	6.07 (1.02)	697	5.30 (1.05)
Rebound (years)	Females	3,225	5.61 (1.16)	647	4.64 (1.10)

**Table 2:** Descriptive statistics of the single nucleotide polymorphisms included in the allelic score.

Chr	Nearest Gene	SNP	Alleles (Effect Allele / Non-effect Allele)	GWAS Effect Size for BMI	Effect Allele Frequency	
					ALSPAC	Raine
1	NEGR1	rs2568958	A/G	0.13	0.5956	0.6218
	TNNI3K	rs1514175	A/G	0.07	0.4249	0.4360
	PTBP2	rs1555543	C/A	0.06	0.5905	0.5942
	SEC16B	rs543874	G/A	0.22	0.2075	0.2021
2	TMEM18	rs2867125	C/T	0.31	0.8325	0.8303
	RBJ, ADCY3, POMC	rs713586	C/T	0.14	0.4888	0.4841
	FANCL	rs887912	T/C	0.1	0.2904	0.2929
	LRP1B	rs2890652	C/T	0.09	0.1669	0.1627
3	CADM2	rs13078807	G/A	0.1	0.2025	0.2089
	ETV5, DGKG, SFRS10	rs7647305	C/T	0.14	0.7924	0.7934
4	SLC39A8	rs13107325	T/C	0.19	0.0764	0.0723
	GNPDA2	rs10938397	G/A	0.18	0.4342	0.4359
5	FLJ35779, HMGCR	rs2112347	T/G	0.1	0.6401	0.6347
	ZNF608	rs4836133	A/C	0.07	0.4949	0.4920
6	TFAP2B	rs987237	G/A	0.13	0.1770	0.1897
9	LRRN6C	rs10968576	G/A	0.11	0.3167	0.3062
	LMX1B	rs867559	G/A	0.24	0.1983	0.1968
11	RPL27A, TUB	rs4929949	C/T	0.06	0.5390	0.5210
	BDNF	rs6265	C/T	0.19	0.8122	0.8119
	MTCH2, NDUFS3, CUGBP1	rs3817334	T/C	0.06	0.4000	0.4213
12	FAIM2	rs7138803	A/G	0.12	0.3592	0.3675
13	MTIF3, GTF3A	rs4771122	G/A	0.09	0.2304	0.2154
14	PRKD1	rs11847697	T/C	0.17	0.0467	0.0414
	NRXN3	rs10150332	C/T	0.13	0.2112	0.2183
15	MAP2K5, LBXCOR1	rs2241423	G/A	0.13	0.7850	0.7699
16	GPRC5B, IQCK	rs12444979	C/T	0.17	0.8620	0.8541
	SH2B1, ATXN2L, TUFM, ATP2A1	rs7359397	T/C	0.15	0.4166	0.3791
	FTO	rs9939609	A/T	0.39	0.3933	0.3835
18	MC4R	rs12970134	A/G	0.23	0.2680	0.2547
19	KCTD15	rs29941	G/A	0.06	0.6848	0.6606
	TMEM160, ZC3H4	rs3810291	A/G	0.09	0.6941	0.6438
	QPCTL, GIPR	rs2287019	C/T	0.15	0.8123	0.8127

### Associations between the allelic score and growth trajectories

**Figure 1:**
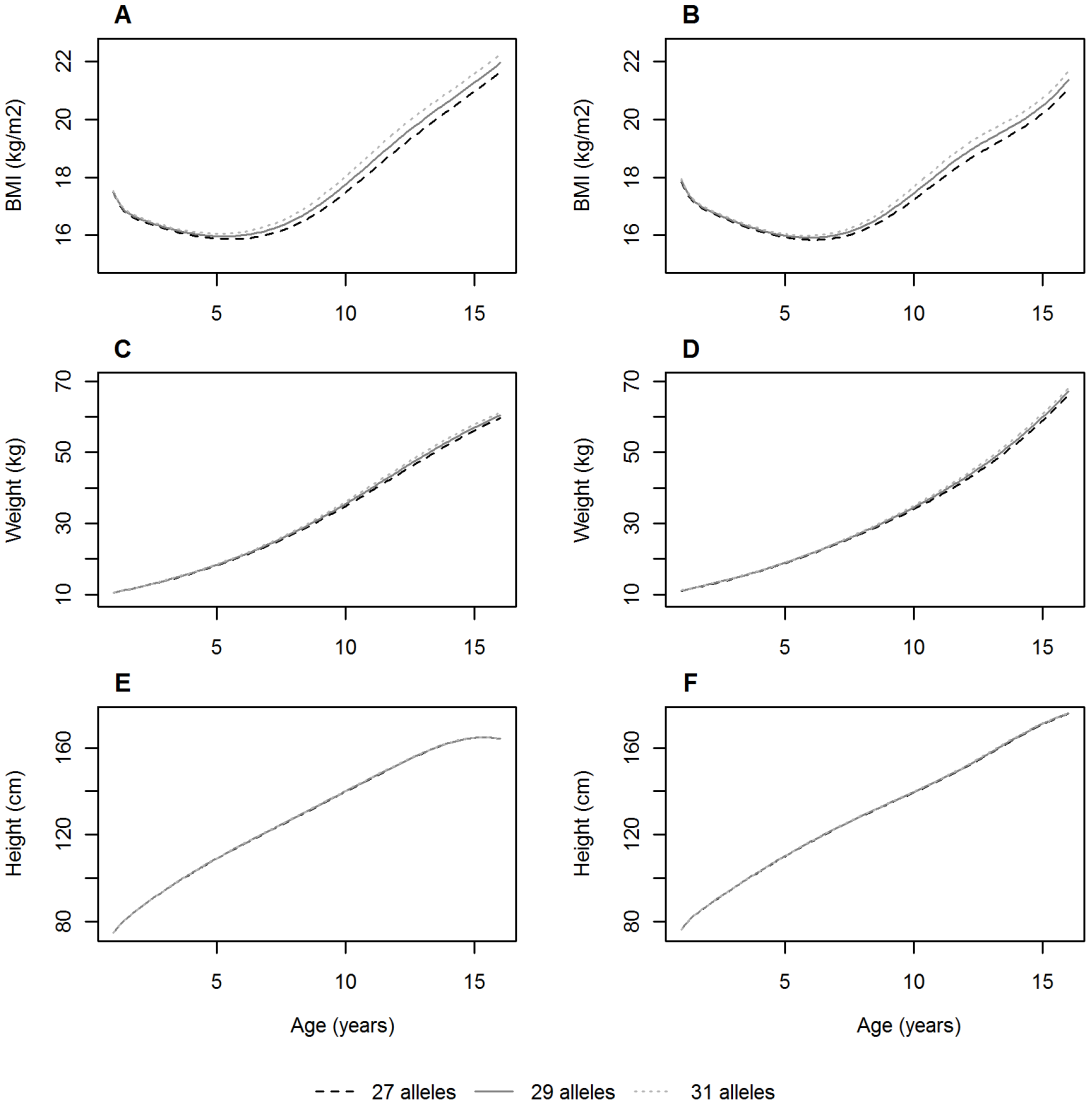
Population average curves for individuals with 27, 29 or 31 BMI risk alleles in females (A, C and E) and males (B, D and F) from the ALSPAC cohort. Predicted population average BMI (A and B), weight (C and D) and height (E and F) trajectories from 1 – 16 years for individuals with 27 (lower quartile), 29 (median), and 31 (upper quartile) BMI risk alleles in the allelic score.

The allelic score was associated with higher mean levels of BMI at the intercept of 8 years (Female: β  =  0.0061 units, P < 0.0001; Male: β  =  0.0044 units, P < 0.0001; Table S1) and faster BMI growth over childhood in both sexes (all age by score interaction P < 0.001). Due to the increasing rate of growth over time, the trajectories of individuals with high and low allelic scores begin together at age one but separate throughout childhood (Figure 1A and 1B). In females, differences in BMI trajectories associated with the allelic score were detectable from just after one year in the ALSPAC cohort and approximately 2.5 years in the Raine cohort; a difference was detected earlier in males, at 1 year in ALSPAC and at 18 months in the Raine cohort.

**Figure 2:**
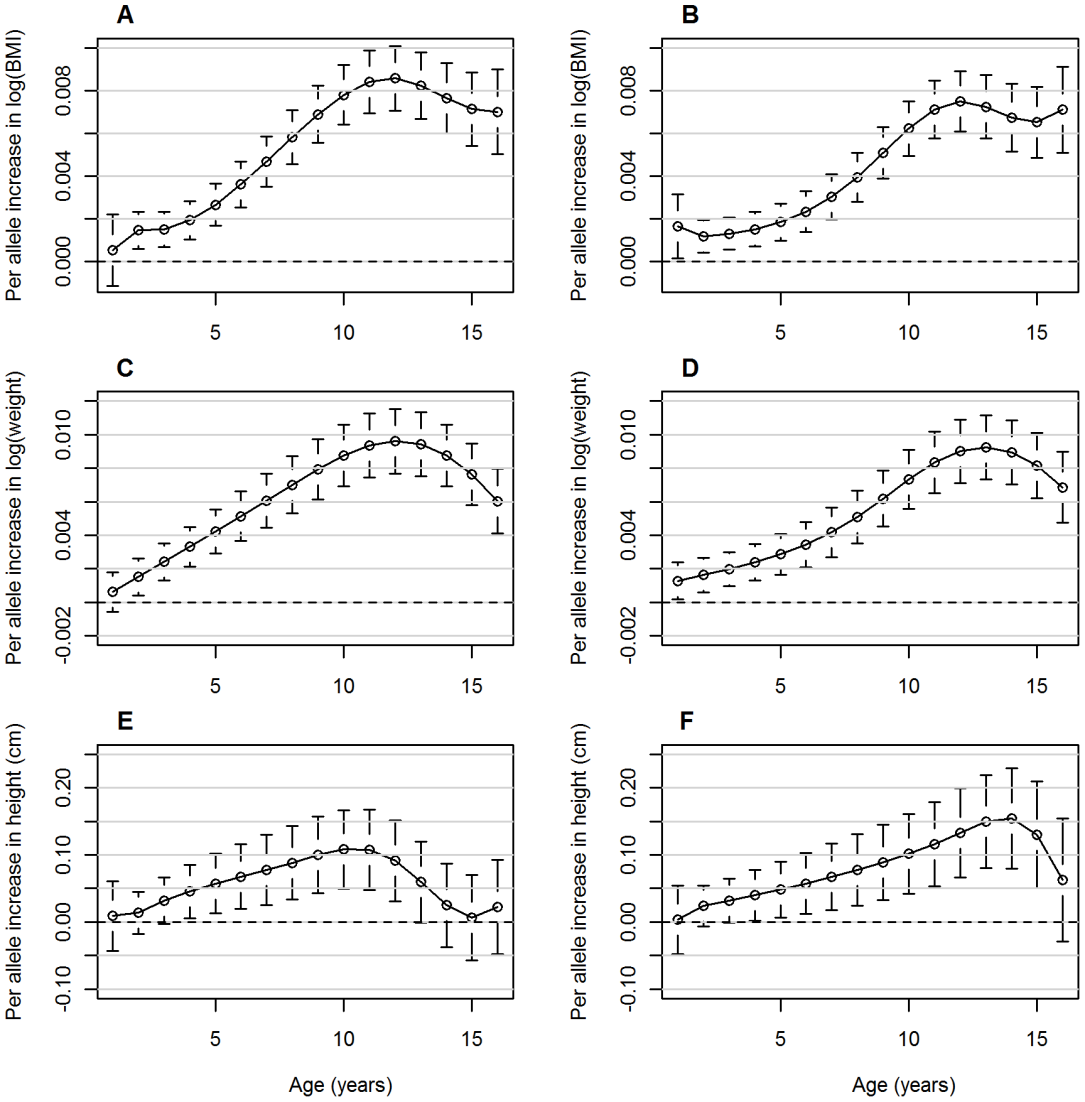
Associations between the allelic score and BMI, weight and height at each follow-up in females (A, C and E) and males (B, D and F) from the ALSPAC cohort. Regression coefficients (95% CI) derived from the longitudinal model at each year of follow-up between 1 and 16 years.

To investigate whether the association of the allelic score with BMI growth over childhood was due to skeletal growth or adiposity, we tested associations between the allelic score and both weight and height measurements. The allelic score was associated with higher weight (Females: β = 0.0073 units, P<0.0001; Males β = 0.0056 units, P<0.0001; Table S1) and faster rates of weight gain over childhood in both males and females (all age by score interaction P<0.001; Figure 1C and 1D). The association with weight was seen earlier in males (by 1 year of age in ALSPAC) than females (around 2 years of age in ALSPAC). The allelic score was associated with increased height in females (β = 0.0949m, P = 0.0002) and males (β = 0.0838m, P = 0.0008) (Table S1) and also displayed evidence for an interaction with age (P<0.001 in ALSPAC, P = 0.001 in Raine females and P = 0.015 in Raine males; Figure 1E and 1F). The effect size of the allelic score on height growth increased over childhood until around 10 years of age in females and slightly later in males and then decreased until it became statistically non-significant (Figure 2C). These results suggest that the association of the allelic score with BMI growth over childhood was due to both skeletal growth and adiposity.

### Associations between the allelic score and birth measures, adiposity peak and adiposity rebound

As expected, females were both lighter and shorter than males at birth (Table 1). The allelic score was not associated with the birth measures in either sex (Table 3). In addition, there was no interaction between the allelic score and gestational age for either weight or length at birth (data not shown).

**Table 3:** Cross-sectional association analysis results for birth measures, BMI and age at adiposity peak (AP) and BMI and age at adiposity rebound (AR) in the ALSPAC and Raine cohorts.

	Females		Males	
	Beta (95% CI)	P-Value	Beta (95% CI)	P-Value
Birth weight (kg)	−0.0004 (−0.0043, 0.0035)	0.8283	0.0026 (−0.0017, 0.0069)	0.2334
Birth length (cm)	−0.0158 (−0.0352, 0.0036)	0.1111	−0.0002 (−0.0190, 0.0186)	0.9840
BMI at AP (kg/m^2^)	0.0163 (0.0079, 0.0248)	0.0002	0.0123 (0.0041, 0.0204)	0.0033
Age at AP (months)	0.0074 (−0.0002, 0.0151)	0.0566	0.0028 (−0.0025, 0.0080)	0.3020
BMI at AR (kg/m^2^)	0.0332 (0.0237, 0.0427)	<0.0001	0.0364 (0.0277, 0.0451)	<0.0001
Age at AR (years)	−0.0362 (−0.0467, −0.0257)	<0.0001	−0.0362 (−0.0450, −0.0274)	<0.0001

The estimated age and BMI at the peak were weakly correlated in females (ρ = 0.08) and males (ρ = −0.30). Later age at adiposity peak was associated with higher BMI at age 15−17 in females but not males. In addition, higher BMI at adiposity peak was associated with higher BMI at age 15−17 years in both sexes. The allelic score was not associated with age of adiposity peak in females or males (Table 3). However, the allelic score was associated with a higher BMI at the peak (Females: β = 0.0163 kg/m^2^, P = 0.0002; Males: β = 0.0123 kg/m^2^, P = 0.0033). Adjustment for age at the peak did not substantively alter the magnitude of the association of the allelic score with BMI at the peak (Females: β = 0.0157 kg/m^2^, P = 0.0003; Males: β = 0.0135 kg/m^2^, P = 0.0007).

Earlier age and higher BMI at the adiposity rebound were both associated with higher BMI at age 15−17 years. The allelic score was associated with an earlier age at the adiposity rebound for females (β = −0.0362years, P<0.0001) and males (β = −0.0362years, P<0.0001) (Table 3). The effect size was attenuated after adjusting for BMI at the rebound (Females: β = −0.0122years, P = 0.0018; Males: β = −0.0096 years, P = 0.0022). The allelic score was also associated with higher BMI at the rebound in females (β = 0.0332 kg/m^2^, P<0.0001) and males (β = 0.0364 kg/m^2^, P<0.0001). Again, the effect size attenuated when adjusting for age at the rebound (Females: β = 0.0094 kg/m^2^, P = 0.0078; Males: β = 0.0109 kg/m^2^, P = 0.0004).

There was a strong positive correlation between BMI at the adiposity peak and the adiposity rebound (Female ρ = 0.65, p<0.0001; Male ρ = 0.59, p<0.0001). BMI at the adiposity rebound explains more of the variation in BMI at age 15−17 (45%) than the BMI at the adiposity peak (10%). Nevertheless, the allelic score remains associated with BMI at the adiposity rebound after adjusting for the BMI at the adiposity peak in both females (β = 0.0171 kg/m^2^, P<0.0001) and males (β = 0.0269 kg/m^2^, P<0.0001).

### Variance explained by the allelic score

**Figure 3:**
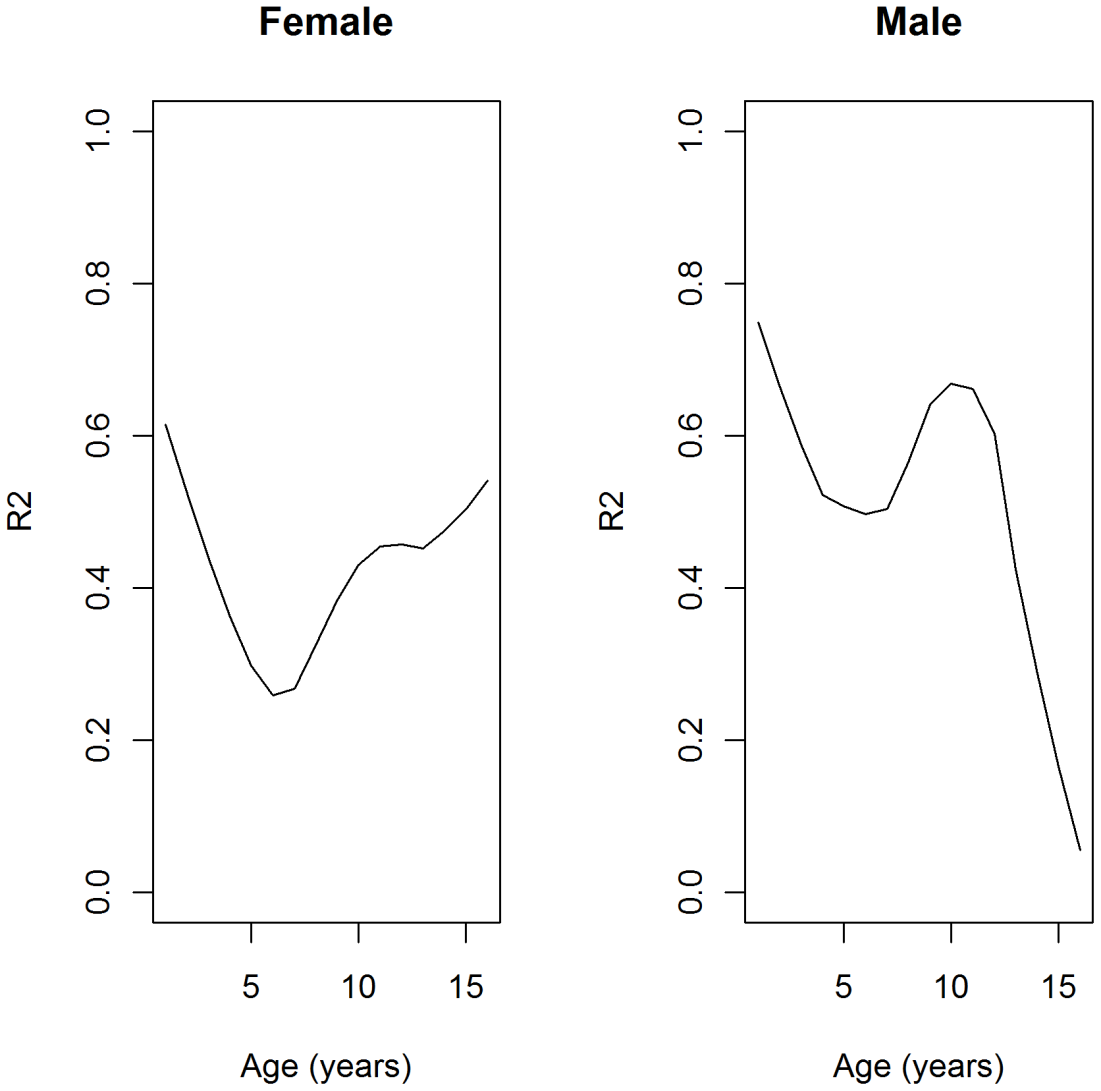
A smooth curve of the estimates from the longitudinal models of the proportion of BMI variation explained (R^2^) at each time point in females and males from the ALSPAC cohort. R^2^ derived from the longitudinal model at each year of follow-up between 1 and 16 years.

We calculated the percentage of variation in BMI explained by the allelic score at each time point in the ALSPAC cohort using the residual sums of squares from the longitudinal BMI growth model [40]. We did not calculate this in the Raine cohort as the sample size was too small for accurate estimates. The allelic score explained 0.58% of the variance in BMI across childhood overall in females and slightly less in males (0.44%) in ALSPAC, but this percentage varied with age (Figure 3). This is approximately a third of the variance in adult BMI explained by these SNPs in the study that identified them [5]. Figure 3 displays the estimates over childhood in females and males.

The allelic score accounted for a similar percentage of BMI at the adiposity peak in both females (0.42%) and males (0.22%). However, for the measures at the adiposity rebound, the allelic score accounts for up to 1−2% of the variation in the two cohorts (Age: 0.87% in ALSPAC females, 2.70% in Raine females, 1.46% in ALSPAC males and 0.89% in Raine males; BMI: 1.01% in ALSPAC females, 1.87% in Raine females, 1.46% in ALSPAC males and 1.14% in Raine males). This is twice as much of the variation in BMI than was able to be accounted for at the time of the adiposity peak or in the overall trajectory.

### Single SNP analyses

In females, five of the 32 individual loci (*RBJ*, *FTO, MC4R, CADM2* and *MTCH2)* reached a Bonferroni significance threshold of 0.0016 in the meta-analysis (Table S2). In males, four of the 32 individual loci (*SEC16B*, *TMEM18, MC4R* and *FTO)* were associated with BMI trajectory at the Bonferroni significance threshold (Table S3). Only FTO and MC4R reached statistical significance in both males and females.

### Sex differences

In analyses combining males and females, there was no evidence for sex interactions for any of the 32 loci after Bonferroni correction; however we report the following result here as an exploratory finding. The sex interaction for the *NRXN3* loci, rs10150332 (including interaction with the spline function), had a P-Value of 0.0039.

## Discussion

We investigated the association of variants in genes known to be associated with increased BMI in adulthood with growth measures over childhood from two extensively characterized longitudinal birth cohorts. Similar to previous studies [13], [14], [15], [16], [17], we have shown that an allelic score of known adult BMI-associated SNPs is not associated with birth measures but is associated with BMI growth throughout childhood and adolescence, weight changes, and also height changes (though with weaker associations). Previous work by Elks et al [13] in the ALSPAC cohort investigated the association of an 8 SNP allelic score with growth trajectories from birth to 11 years of age. We have extended their work by including an additional cohort, and by increasing the age period over which the trajectories are examined and the number of SNPs investigated. By extending the age range, we have shown that the association between the allelic score and weight changes increases in magnitude with age, whereas the association of the allelic score with height growth stops after the onset of puberty. Belsky et al [15] are the only other investigators to look at an allelic score using the same set of SNPs; our conclusions are similar to theirs in terms of the growth trajectories throughout childhood, but we extend their work by i) having more detailed early growth measurements, enabling us to show that the allelic score starts to be associated with growth trajectories at an early age and to assess associations between the allelic score and the adiposity peak in infancy, and ii) some exploratory findings regarding sex specific genes effecting BMI growth. The GIANT consortium found a SNP 30,000 bp upstream from the *RBJ* loci and a SNP in the *MC4R* gene to be associated with adult height [42], but the full functional relevance of the 32 loci, and which of them affect height, fat accumulation or both, is not yet understood, and our study does not have sufficient power to address this. A useful extension to the current study would be to investigate whether any of the individual SNPs in the allelic score largely influence child height growth rather than weight; however a larger sample size would be required to consider this.

Although the effect sizes presented appear relatively small, they are consistent with those previously reported in the adult studies. At age 15, an increase of one BMI risk allele increases BMI by approximately 0.15 kg/m^2^, which is equivalent to some of the mid-range effect sizes from adult GWAS studies as reported in Table 2. It is widely known that the genetic basis of obesity is still largely unknown, with only 1.45% of the variation in BMI due to genetics having been described [5]; however, this study sheds more light on the mechanisms behind how these genetic variants influence childhood growth, rather than describing particularly large effects sizes from any individual SNP.

Our results suggest that known adult BMI increasing alleles have a detectable effect on childhood growth as early as one year. In addition, we investigated the association between the allelic score and features of the growth curve thought to be associated with later obesity and cardiovascular health [23], [24], [43], [44], [45]; the allelic score was positively associated with higher BMI at the adiposity peak, but only weakly associated with age at adiposity peak. This contrasts the findings for the association between the *FTO* gene and adiposity peak shown in the Northern Finnish Birth Cohort from 1966 [25], where the age but not BMI at adiposity peak was associated with the *FTO* variant; however, subsequent analysis in this cohort as part of a meta-analysis showed the association was not statistically significant [17]. The explanation for these differences are unclear; both of the cohorts investigated had limited data available in the first few years of life, and although data availability was greater than in previous studies and we were able to estimate the emergence of the genetic association and the parameters around the adiposity peak, it would be beneficial to replicate this finding in cohorts with more regular measurements in early infancy. Likewise, we saw differences in the timing of the adiposity rebound between the ALSPAC and Raine cohorts, with an earlier rebound being found in the Raine cohort. This could be due to the lack of data between three and five and a half years where the rebound is expected to occur. In contrast, the ALSPAC cohort had an adequate number of measurements throughout the adiposity rebound period although a portion of them came from parental report questionnaires which have been shown to be less accurate than the clinic measures [46]. Therefore, the precision of the estimate for the BMI and age at the adiposity rebound is very similar between the two cohorts, as seen by the standard deviations in Table 1. In addition, we do not believe this has influenced the genetic results as the effect sizes of the allelic score were similar between the ALSPAC and Raine cohorts for both the age and BMI at the adiposity rebound (data not shown).

Previous studies investigating the association between adult BMI associated SNPs and childhood growth adjusted their analyses for sex [13], [14], [15], [16], [17]; only Hardy et al [16] tested for a sex interaction and found it to be non-significant. We detected a statistically significant sex interaction for the allelic score, so conducted sex specific analyses. We found that the allelic score begins to be associated with BMI and weight earlier in males than females, but around the same age for height. Furthermore, other than the *FTO* and *MC4R* SNPs, we found different genes associated with childhood BMI trajectory in males and females. However, these differences could not be replicated in the formal interaction analysis and therefore further investigation in larger sample sizes is required to confirm this observation. Our findings provide additional evidence that there may be different, but partially overlapping, genes that contribute to the body shape of males and females from early childhood.

In conclusion, we have conducted an association analysis in a large childhood population to investigate the effect of known adult genetic determinants of BMI on childhood growth trajectory. We have shown that the genetic effect begins very early in life, which is consistent with the life course epidemiology hypotheses – the determinants of adult susceptibility to obesity begin in early childhood and develop over the life course.

## Supporting Information

Table S1 Longitudinal allelic score association analysis results for BMI, weight and height in ALSPAC and Raine, in addition to the meta-analysis summary
(XLSX)

Table S2 Longitudinal association analysis results for each of the 32 BMI SNPs against BMI, weight and height in females from ALSPAC and Raine, in addition to the meta-analysis summary
(XLSX)

Table S3 Longitudinal association analysis results for each of the 32 BMI SNPs against BMI, weight and height in males from ALSPAC and Raine, in addition to the meta-analysis summary
(XLSX)

Methods S1 Additional information regarding the collection of phenotypic measurements and genotyping methods in the ALSPAC and Raine cohorts. Furthermore, additional details regarding the longitudinal modelling and derivation of growth phenotypes are provided.
(DOC)
