# Supplementary material for: Association of a Body Mass Index Genetic Risk Score with Growth throughout Childhood and Adolescence
Source: PLoS One. 2013 Nov 11;8(11):e79547. doi: 10.1371/journal.pone.0079547 (PMC3823612; doi:10.1371/journal.pone.0079547)
Supplement: Methods S1 — Additional information regarding the collection of phenotypic measurements and genotyping methods in the ALSPAC and Raine cohorts. Furthermore, additional details regarding the longitudinal modelling and derivation of growth phenotypes are provided. (DOC) [file pone.0079547.s004.doc]

Association of a Body Mass Index Genetic Risk Score with Growth throughout Childhood and Adolescence

Nicole M. Warrington*^1,2^, Laura D. Howe*^3^, Yan Yan Wu^2^, Nicholas J. Timpson^3^, Kate Tilling^4^, Craig E. Pennell^1^, John Newnham^1^, George Davey-Smith^3^, Lyle J Palmer^2,5^, Lawrence J Beilin^6^, Stephen J. Lye^2^, Debbie A. Lawlor^3^, Laurent Briollais^2†^

**Supplementary Methods:**

**Measurements:**

*ALSPAC:* Birth length (crown-heel) was measured by ALSPAC staff who visited newborns soon after birth (median 1 day, range 1-14 days), using a Harpenden Neonatometer (Holtain Ltd). Birth weight was extracted from medical records. Gestational age was obtained from obstetric medical records, as recorded by health care professionals, who used data from the woman’s reported last menstrual period, obstetric assessment during the antenatal period, ultrasound assessment and paediatric assessment at birth; at the time that this cohort was established routine early pregnancy data scans were not conducted and it is likely that only a minority had gestational age determined by ultrasound scan. From birth to five years, length and weight measurements were extracted from health visitor records, which form part of standard child care in the UK. In this cohort we had up to four measurements taken on average at six weeks, 10, 21, and 48 months of age. For a random 10% of the cohort, we also have length/height measurements from eight research clinic visits, held between the ages of four months and five years of age. From age seven years upwards, all children were invited to clinics at intervals of approximately one year. Details of the measuring equipment used in the clinics is described elsewhere . In addition, parent-reported child height and weights were also available from the questionnaires (27% of measures). BMI was calculated from the weight and height measurements (median 9 measures per person, interquartile range 5-12, range 1-29 measurements), with a total of 68,862 BMI measures.

*Raine:* Birth length was measured between 24 and 72 hours post birth using a Harpenden Neonatometer to the nearest 0.1cm. Birth weight was measured in the hospital at birth. Gestational age was based on the date of the last menstrual period unless there was discordance with ultrasound biometry at the dating scan by greater than 7 days; if there was a discordance the gestational age was based on the dating scan . Weight and height were collected at the follow-up clinics at average ages of 1, 2, 3, 6, 8, 10, 14 and 17 years by trained members of the research team ; weight was measured using a Wedderburn Digital Chair Scale to the nearest 100g with children dressed in running shorts and a singlet top and height was measured to the nearest 0.1cm with a Holtain Stadiometer. BMI was calculated from the weight and height measurements (median 6 measures per person, interquartile range 5-7, range 1-8 measurements), with a total of 8,687 BMI measures.

**Genotyping and allelic score:**

ALSPAC children were genotyped using the Illumina HumanHap550 quad genome-wide SNP genotyping platform by 23andMe subcontracting the Wellcome Trust Sanger Institute, Cambridge, UK and the Laboratory Corporation of America, Burlington, NC, US. Raine children were genotyped on an Illumina 660 Quad Array at the Centre for Applied Genomics, Toronto, Canada. Standard quality control methods were performed in each sample separately and have been previously described . Genotypic data were imputed using MACH v1.0.16 for the all 22 autosomes with the CEU samples from HapMap Phase2 used as a reference panel.

For both cohorts, single nucleotide polymorphisms (SNPs) were removed if the minor allele frequency (MAF) was < 1%, the call rate was < 95% or the P-value from an exact test of Hardy-Weinberg equilibrium was <5.7 x 10^-7^. Individual samples were excluded on the basis of incorrect gender assignment, minimal or excessive heterozygosity, high levels of missingness and cryptic relatedness (16% and 6% of those genotyped in ALSPAC and Raine respectively).

**Longitudinal Modelling and derivation of growth parameters:**

The effect estimates of the allelic score on BMI over time presented in Figure 2 were estimated using the General Linear Hypothesis approach . This approach is based on the normal approximation for maximum likelihood estimators using the estimated variance-covariance matrix. The hypothesis can be specified through a constant matrix *L* to be matched with the fixed effects of the model such that
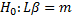
 where the *m* are the hypothesized values. The estimates of fixed effects
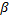
, asymptotically follow a multivariate normal distribution
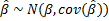
 by the Central Limit Theorem such that the linear form also asymptotically follows a multivariate normal distribution:


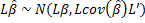


Thus the 95% confidence interval and corresponding P-value for the hypothesized value can be obtained accordingly.

Age and BMI at adiposity rebound were derived by setting the first derivative of the fixed and random effects from the BMI model between 2 and 8 years of age for each individual to zero (i.e. the minimum point in the curve). In addition, a second model was fit in the ALSPAC cohort only, between birth and 5 years to derive the adiposity peak, with each individual required to have greater than 2 measures throughout this period, similar to Silverwood et al . BMI measurements after 5 years were excluded to avoid the period of adiposity rebound. This model had knot points at ages 1 and 2.5 years and a cubic slope between each. Adiposity peak was derived by setting the first derivative of the fixed and random effects between birth and 2.5 years to zero. Raine does not have enough repeated measurements between birth and one year of age to justify calculating the adiposity peak.

Given that growth curves differ greatly between males and females, particularly around puberty, and because different genes may influence the timing of growth spurts in males and females, we investigated whether there was an effect of sex over the time period. Preliminary analysis showed evidence for a sex-age interaction in both cohorts (likelihood-ratio test [LRT] P < 0.0001 in both cohorts); therefore all analyses were conducted stratified for sex.

**References:**

1. Howe LD, Tilling K, Benfield L, et al. Changes in ponderal index and body mass index across childhood and their associations with fat mass and cardiovascular risk factors at age 15. *PLoS One* 2010;**5**(12): e15186.

2. Evans S, Newnham J, MacDonald W, Hall C. Characterisation of the possible effect on birthweight following frequent prenatal ultrasound examinations. *Early Hum Dev* 1996 Jul 19;**45**(3): 203-14.

3. Huang RC, Burke V, Newnham JP, et al. Perinatal and childhood origins of cardiovascular disease. *Int J Obes Res* 2006 May 23.

4. Warrington NM, Wu YY, Pennell CE, et al. Modelling BMI Trajectories in Children for Genetic Association Studies. *PLoS One* 2013;**8**(1): e53897.

5. McDonald L. Tests for the General Linear Hypothesis Under the Multiple Design Multivariate Linear Model. *The Annals of Statistics* 1975;**3**(2): 461-6.

6. Silverwood RJ, De Stavola BL, Cole TJ, Leon DA. BMI peak in infancy as a predictor for later BMI in the Uppsala Family Study. *Int J Obes (Lond)* 2009 Aug;**33**(8): 929-37.
